# Predicting Hospital-Acquired Infections by Scoring System with Simple Parameters

**DOI:** 10.1371/journal.pone.0023137

**Published:** 2011-08-24

**Authors:** Ying-Jui Chang, Min-Li Yeh, Yu-Chuan Li, Chien-Yeh Hsu, Chao-Cheng Lin, Meng-Shiuan Hsu, Wen-Ta Chiu

**Affiliations:** 1 Graduate Institute of Medical Science, College of Medicine, Taipei Medical University, Taipei, Taiwan; 2 Department of Dermatology, Far Eastern Memorial Hospital, New Taipei, Taiwan; 3 Department of Nursing, Oriental Institute of Technology, New Taipei, Taiwan; 4 Department of Dermatology, Taipei Medical University Wan Fang Hospital, Taipei, Taiwan; 5 Graduate Institute of Biomedical Informatics, College of Medical Science and Technology, Taipei Medical University, Taipei, Taiwan; 6 Center of Excellence for Cancer Research (CECR), Taipei Medical University, Taipei, Taiwan; 7 Department of Psychiatry, National Taiwan University Hospital, Taipei, Taiwan; 8 Section of Infectious Disease, Department of Internal Medicine, Far Eastern Memorial Hospital, New Taipei, Taiwan; 9 Graduate Institute of Injury Prevention and Control, Taipei Medical University, Taipei, Taiwan; University of Swansea, United Kingdom

## Abstract

**Background:**

Hospital-acquired infections (HAI) are associated with increased attributable morbidity, mortality, prolonged hospitalization, and economic costs. A simple, reliable prediction model for HAI has great clinical relevance. The objective of this study is to develop a scoring system to predict HAI that was derived from Logistic Regression (LR) and validated by Artificial Neural Networks (ANN) simultaneously.

**Methodology/Principal Findings:**

A total of 476 patients from all the 806 HAI inpatients were included for the study between 2004 and 2005. A sample of 1,376 non-HAI inpatients was randomly drawn from all the admitted patients in the same period of time as the control group. External validation of 2,500 patients was abstracted from another academic teaching center. Sixteen variables were extracted from the Electronic Health Records (EHR) and fed into ANN and LR models. With stepwise selection, the following seven variables were identified by LR models as statistically significant: Foley catheterization, central venous catheterization, arterial line, nasogastric tube, hemodialysis, stress ulcer prophylaxes and systemic glucocorticosteroids. Both ANN and LR models displayed excellent discrimination (area under the receiver operating characteristic curve [AUC]: 0.964 versus 0.969, p = 0.507) to identify infection in internal validation. During external validation, high AUC was obtained from both models (AUC: 0.850 versus 0.870, p = 0.447). The scoring system also performed extremely well in the internal (AUC: 0.965) and external (AUC: 0.871) validations.

**Conclusions:**

We developed a scoring system to predict HAI with simple parameters validated with ANN and LR models. Armed with this scoring system, infectious disease specialists can more efficiently identify patients at high risk for HAI during hospitalization. Further, using parameters either by observation of medical devices used or data obtained from EHR also provided good prediction outcome that can be utilized in different clinical settings.

## Introduction

Hospital-acquired infections (HAI), also known as Nosocomial Infections (NI) or health-associated infections, are associated with increased attributable morbidity, mortality, prolonged hospitalization, and economic costs [1], [2]. The exact prevalence rate of HAI varies from country to country, the clinical settings (e.g. general wards vs. intensive-care units, ICU) disciplines (e.g. medical vs. surgical) and anatomical sites (e.g. bloodstream infection, respiratory infection, urinary tract infection, surgical site infection and soft tissue infection, etc). The Study on the Efficacy of Nosocomial Infection Control (SENIC) project estimated that approximately 2.1 million nosocomial infections occurs annually among 37.7 million admissions in US and the mortality rate reported to be 77,000, associated with nosocomial infections [3], [4]. The underlying causes are frequent invasive procedures, multiple drug therapies and complicated diseases. The ICU has higher prevalence rates of nosocomial infections [5], ranging from 31.5% to 82.4% in bloodstream infections [6], and is at risk of mortality.

Hospital-acquired infections is defined as an infection not present or incubating at the time of admission to hospital or other health-care facility [7], and the diagnostic time frame is clearly dependent on the incubation period of the specific infection; 48 to 72 hours post-admission is generally regarded as indicative of HAIs [8].

In addition to the association with morbidity and mortality, HAIs are frequently associated with drug-resistant microorganisms, such as methicillin-resistant Staphylococcus aureus (MRSA) and extended spectrum β-lactamase (ESBL)-producing gram-negative bacteria, which are increasingly prevalent in the hospitals and the communities [8]. Hospital-acquired infections can affect on any part or organ of the body. Vincent et al [5] observed more frequent cases of upper and lower respiratory tract infections, followed by urinary tract infections and bloodstream infections. Seven risk factors for ICU-acquired infection were identified: increased duration of ICU stay (>48 hours), mechanical ventilation, diagnosis of trauma, central venous, pulmonary artery, and urinary catheterization, and stress ulcer prophylaxes. ICU-acquired pneumonia (odds ratio [OR], 1.91; 95% confidence interval [CI], 1.6–2.29), clinical sepsis (OR, 3.50; 95% CI, 1.71–7.18), and bloodstream infection (OR, 1.73; 95% CI, 1.25–2.41) increased the risk of ICU death.

There are several predisposing factors contributing HAI. It is observed that factors are associated with either an increased risk of colonization or with decreased host defense, which could be divided as: those related to underlying health impairment such as age, smoking habits, diabetes; those related to the acute disease process such as surgery or burns; those related to the use of invasive procedures or other mode of treatment [1], [5], [8], [9], [10], [11].

Advancement of medical science and technology help to make devices, which developed to improve patient care, both in diagnostic and therapeutic purposes. However, such invasive devices increase the survival for patients yet put them at high risk for infection. In critically ill patient population, 97% of cases of urinary tract infection are due to catheterization, 87% of cases of bloodstream infection of a central line and 83% of cases of pneumonia are associated with mechanical ventilation [11]. The devices have been regarded as important factors in predisposing HAIs.

To evaluate the relationship between risk factors and HAI, there are several published statistic and mathematical methods. Logistic Regression (LR) is one of the well known method, other methods including multi-state model [12], and artificial neural networks (ANN) are used for prediction purpose [13], [14].

Among the mathematical and statistical modeling techniques used in clinical decision support system, ANN is frequently used in recent studies. These systems in their most basic implementation consist of a layer of input variables, connected to an intermediate layer of derived variables (a ‘hidden’ layer), and then to the final output prediction. Processing of multiple events occurs in the hidden layer, with final results passed to the output layer. The connections between these neurons represent mathematical functions that propagate the modified ‘impulse’ to the next neuron. By changing the transfer functions and the associated parameters, this constructed neural network adapts itself to the pattern of the input variables and eventually generates numbers that iteratively solves to values of the designated output variables.

Currently, ANN and LR are the most widely used models in biomedicine, as Dreiseitl and Ohno-Machado reviewed in 2002, there were 28,500 publications for LR and 8,500 for ANN indexed in MEDLINE [15], and the number is believed to be increasing. According to the discriminatory power, there exist no difference between ANN and LR [15].

The relevant patient clinical data collection is another task for statistical analysis. Previously, chart review is the only way to fulfill this work that is laborious and time consuming. As the progress of hospital information systems, the electronic health records (EHR) or computerized patient records (CPR) are widely used in Taiwan. In 2005, the EHR coverage is observed to be 44% and 55% in clinics and hospitals respectively, with up to 78% coverage in medical centers or university hospitals [16]. For reimbursement's purpose, each invasive/noninvasive procedure with matched instruments/materials, medication, physician order and action time is electronically recorded so that all procedure carried out during admission is not misplaced, otherwise the national health insurance bureau can deny reimbursement to the hospitals. Due to the above mentioned factors EHR offers the platform to provide non-clinical patient data. If the clinical data is collected automatically from EHR, the data collection task can be easily completed. With this advantage, statistical analysis can be conveyed in a timesaving way, so that patient data is immediately available at any time so as to assist in optimal clinical decision making even upon admission. World-wide increased use of EHR in identifying risk factors for HAI from residential information [17] and applying administrative coding data as a surveillance tool in HAI [18], [19] have been evaluated. However, according to best of the knowledge, there is no study using abstracted data generated from EHR to predict the outcome of risk assessment.

The medical scoring systems are widely used to predict risk of morbidity or mortality and to evaluate outcome in patients with certain illness. The first system of this kind was the APGAR score in assessing the vitality of the newborn [20]. There are 4 categories of medical scoring systems [21]: 1. General risk-prognostication (severity of illness) scores such as APACHE (Acute Physiology and Chronic Health Evaluation); 2. Disease- and organ- specific prognostic scores such as GCS (Glasgow coma scale); 3. Trauma scores such as traumatic brain injury score [22]; and 4. Organ dysfunction (failure) scores such as SOFA and MODS. The scoring systems have also been included in other more complex systems. The value of such scoring systems is to provide a simple predictive tool with certain relevant factors for clinical use.

Up until the present, there exists no such scoring system for HAI. A simple, reliable predictive model for HAI is of great clinical relevance. The primary goal of this study is to construct a scoring system to predict patients at risk for HAI, and to validate the system by ANN and LR that will be the foundation for computation in the future.

## Methods

### Ethics Statement

This study was approved by the Institutional Review Board of Taipei Medical University Wan Fang Hospital.

### Study Population

The EHR data from Taipei Medical University Wan Fang Hospital, an 800-bed academic teaching center, were used to select inpatients with HAI. During 2004 to 2005, there were 806 patients with 1,297 records of HAI who met the diagnostic criteria for Centers for Disease Control and Prevention of the United States, and were enrolled and verified by the infection control unit that included full-time nurses, medical technicians and infectious disease specialists. The final enrollment of the HAI patients, taking urinary tract infection for instance, was determined not only by microbiological results but also patient's clinical conditions such as fever, pyuria, and other laboratory data relevant to the diagnosis of “infection” instead of colonization. The relevant clinical data was manually recorded in the electronic form. Non-HAI cases were sampled from a total of 69,032 patients from EHR in the same period of time for control group. Only patients with first episode of infection were considered, and excluded were patients younger than 16 years of age or older than 80 years and more than 60 days of hospital stay. There were 1,852 records with 476 in infection and 1,376 in control groups respectively. All the patient-specific characteristics such as chart number, name and ID were censored and recorded. The Institutional Review Board of Taipei Medical University Wan Fang Hospital waived the informed consent requirement because the data were analyzed anonymously.

### Data preparation

All the variables used in control group were generated from EHR including basic demographic data, duration before infection, underlying health status, acute disease progress, invasive procedures and modes of treatment. Taking the advantage of EHR, data collection becomes easy and can be classified for statistical purpose immediately. For example, there are ICD-9-CM codes for diagnosis and procedure codes for chest tube insertion, we applied simple query to get the information immediately. EHR is still limited for information collection, such as vital signs, adverse events of medications and procedures, and even patients' complaints and laboratory testing reports if patient-specific health records or history progress notes are not well constructed. At the time of data collection during period of 2004–2005, such effective system was not available. Discussion with infection specialists for reflecting the clinical situations was done then we calculated the number of diagnosis at admission represented as complexity of disease, opted for general anesthesia for those who had major surgical procedures and interventions, advised for hemodialysis as the predictors of underlying healthy status. Interventional procedures or devices used, including endotracheal tube and tracheostomy, nasogastric (NG) tube, arterial line and central venous catheterization (CVC), Foley catheterization, and draining tubes implantation (chest tube, draining tube, double-lumen tube … etc) were recorded. The medications such as systemic glucocorticosteroids used for more than 5 days; non-steroid anti-inflammatory drugs (NSAID), stress ulcer prophylaxes (H2 antagonists, sucralfate, and proton pump inhibitors) and chemotherapeutic agents for more than 3 days were also collected. There are 16 variables including 2 demographic, 3 underlying health status-related, 7 procedural, and 4 therapeutical variables (table 1). The characters of demographic data and coding for variables with univariate analyses for both groups are shown in table 2. The major outcome is infectious or non-infectious.

**Table 1:** Variables Used for Statistical Analysis.

Category	Variables	Remark
Demographic	Age	
	Gender	
Underlying health status	Diagnosis number at admission	Represented as complexity of disease
	General anesthesia	Major surgical procedures and interventions
	Hemodialysis	Underlying healthy status
Procedural	Arterial line	
	Central venous catheterization	
	Endotracheal intubation	
	Tracheostomy	
	Nasogastric tube	
	Foley catheterization	
	Draining tubes	Chest tube, draining tube, double-lumen tube…etc.
Therapeutical	Chemotherapeutic agents	Used for more than 3 days
	Systemic Glucocorticosteroids	Used for more than 5 days
	Stress ulcer prophylaxes	H2 antagonists, sucralfate, and proton pump inhibitors used for more than 3 days
	Non-steroid anti-inflammatory drugs	Used for more than 3 days

**Table 2:** Univariate Analyses for Demographic and Clinical Data of Infection and Non-Infection Sets (N = 1,852).

		Infection	Non-infection
Variables^a^	Coding	(N = 476)	(N = 1,376)
Age, years^b^^,^ *		65.32±13.40	51.82±18.55
Diagnosis numbers at admission^b^^,^ *		1.67±1.01	1.41±0.76
Gender*	Male	274 (57.6%)	685 (49.8%)
	Female	202 (42.4%)	691 (50.2%)
General anesthesia*	Yes	222 (46.6%)	368 (26.7%)
	No	254 (53.4%)	1,008 (73.3%)
Hemodialysis*	Yes	75 (17.3%)	42 (3.1%)
	No	401 (82.7%)	1,334 (96.9%)
Arterial line*	Yes	216 (45.4%)	59 (4.3%)
	No	260 (54.6%)	1317 (95.7%)
CVC*	Yes	296 (62.2%)	89 (6.5%)
	No	180 (37.8%)	1,287 (93.5%)
Endotracheal intubation*	Yes	378 (79.4%)	133 (9.7%)
	No	98 (20.6%)	1,243 (90.3%)
Tracheostomy*	Yes	107 (22.5%)	17 (1.2%)
	No	369 (77.5%)	1,359 (98.8%)
NG tube*	Yes	420 (88.2%)	151 (11.0%)
	No	56 (11.8%)	1,225 (89.0%)
Foley catheterization*	Yes	355 (74.6%)	202 (19.8%)
	No	121 (25.4%)	1,104 (80.2%)
Draining tubes*	Yes	58 (12.2%)	22 (1.6%)
	No	418 (87.8%)	1,354 (98.4%)
Chemotherapy*	Yes	24 (5.0%)	19 (1.4%)
	No	452 (95.0%)	1,357 (98.6%)
Systemic Glucocorticosteroids*	Yes	143 (30.0%)	42 (3.1%)
	No	333 (70.0%)	1,334 (96.9%)
Stress ulcer prophylaxes*	Yes	331 (69.5%)	130 (9.4%)
	No	145 (30.5%)	1,246 (90.6%)
NSAID	Yes	136 (28.6%)	430 (31.3%)
	No	340 (71.4%)	946 (68.8%)

a Statistics of each variable between infection and non-infection sets,

b Mean±SD,

*p<0.05.

Abbreviations: CVC = central venous catheter, NG = nasogastric, NSAID: non-steroid anti-inflammatory drug.

The set of data obtained from EHR was randomly divided into three groups: training set, selection set and test set. The training set with 927 cases, as approximately 50% of the entire cohort, was used to build LR and ANN models. The selection set of 464 cases was used for ANN modeling (25% of the cohort) in avoiding overfitting and as an early stopping method [23]; and the test set represents 461 cases (25% of the cohort) for internal validation.

### Logistic Regression Analysis

Multiple logistic regression analysis was first performed using the same training set of 927 cases as the ANN analysis for maximum likelihood estimation. Although the LR does not involve “training”, we used this training set to refer to the portion of data used to derive the regression equation [23]. A backward stepwise algorithm was used to construct the LR model and estimate the coefficient (β) of the variables. The likelihood ratio test was used to assess the covariate-adjusted *p* value. Based on the result, the probability of infection was estimated using the logistic equation.

To obtain the most optimal prediction with few variables, we applied a “variable rotation” method in building a reasonable model in order to fit the different clinical settings regarding the ease of information retrieval. First, variables relevant to HAI from the literatures or with higher likelihood ratio, such as Foley catheter, CVC, arterial line and NG tube, was excluded individually or combined in groups from first LR model, and then block entry of variables was used for further analysis. The definition and content of the groups are shown in table 3. The performance of each LR model was compared by the area under receiver operating characteristic (ROC) curve [24].

**Table 3:** Definition of Variable Groups for Analysis.

Setting	Variables	Remark
Group 1	All 7 variables	Selected by final LR model
Group 2	Foley catheter, NG tube and steroids	High odds ratio variables
Group 3	Foley catheter, CVC, arterial line and NG tube	Medical devices
Group 4	CVC, arterial line and stress ulcer prophylaxes	Low odds ratio variables
Group 5	Hemodialysis, stress ulcer prophylaxes and steroids	Underlying condition and medications

Abbreviations: CVC = central venous catheter, NG = nasogastric, LR = logistic regression.

The models were then applied, using the statistically significant variables obtained, to detect the cases of infection in the internal validation set of 461 patients as in the test set.

### Artificial Neural Networks

The ANN model was constructed by several architectures of feedforward networks, including linear, multilayer perceptron and radial basis function networks. The networks consisted of one input layer with several input nodes (16), a hidden layer, and an output layer. The number of hidden nodes to be used is not clear and there is not any well-established protocol existing to determine the numbers. The output layer represents the prediction of infection was set to be of a categorical value of 1 and non-infection was 0. The training technique was set as back-propagation and conjugate gradient descent algorithms, which adjusts the internal parameters of the network over repeated training cycles to reduce the overall error. We applied the same steps used in LR for ANN modeling with the three data sets. In the comparison of discrimination ability with LR, we used the values of probability in training set, which was optimally predicted by selection set in the modeling process; and used the values of probability in same model for internal validation.

### Scoring System

After completing LR, a shrinking power transformation was then applied. This procedure uses the log transformation to reduce the influence of extreme score values on the prediction. The same variable selection procedures used in LR were also applied in developing this system. The cut-off points for each variable group were determined by ROC.

### External Validation

In order to provide an unbiased estimate of the discrimination and calibration of the models, these values should be calculated from external data set. All admitted patient records from November 2010 from a different 1,200-bed academic tertiary teaching center were used for external validation of final ANN, LR and scoring models. Using the excluding criteria defined previously, 2,500 records were used as an external validation data set. The predictive performance of our models was examined for the new data set.

### Statistical Analysis

Univariate analyses were performed to compare the differences of demographic and predictive variables between infection and control groups. Chi-square testing was used for categorical data and Student's t-testing for continuous data while statistical significance level was defined as *p*<0.05. Mean values (±SD) were used to present continuous variables and frequencies were used to present categorical variables. The statistical software used for LR was SPSS for windows (Version 17.0, SPSS Inc, Chicago, Illinois, USA). ANN was conducted by STATISTICA Neural Networks (Release 7.0E, StatSoft Inc, Tulsa, OK, USA). The areas under receiver operating characteristic curve (AUC) were calculated and compared using MedCalc for windows, version 10.2.0.0 (MedCalc Software, Meriakerke, Belgium).

## Results

Out of 1,852 patients, 893 (48.2%) were female patients with the mean age of 55.29±18.35 years (range 17 years–80 years); Mean diagnosis numbers at admission was 1.48±0.838 (range 0–5). Table 2 summarized the demographic clinical characteristics of infection and non-infection groups. The patients with HAIs were found to be older and predominantly male, and have significantly increased number of diagnosis at the time of admission, hemodialysis, devices used such as arterial lines, CVCs, endotracheal intubations and tracheostomy, NG and other draining tubes, Foley catheters, and treatment modalities as chemotherapy, systemic steroids and ulcer prophylaxes than those without infection. There was no statistical significance between two groups with respect to NSAID per se. These parameters were used to establish LR and ANN models.

### Detection of Infection by Logistic Regression

We first analyzed the variables that would be useful to detect infection and 7 variables were included in the final LR model selected by stepwise method which is shown in table 4. The optimal cut point (Youden's index) for predicted values was 0.20. The performances of LR of Group 1 both for training set (accuracy: 91.05%; sensitivity: 93.7%; specificity: 91.0%) and internal validation (accuracy: 91.54%; sensitivity: 92.44%; specificity: 91.52%) were excellent. Then we applied “variable rotation” methods in comparison with different variable groups, with particular focus on the presence or absence of medical devices in determining the acceptable models. Using medical devices as variables only (i.e. Group 3), we displayed comparatively good performances with high accuracy (90.40% in training set and 91.76% in internal validation), the mean values of AUC were 0.953±0.010 and 0.959±0.013 for training and internal validation sets respectively. Finally, we found that using only three variables representing underlying condition and medications (i.e. Group 5) can also give satisfactory prediction rates in internal validation (accuracy: 85.33%; sensitivity: 71.43%; specificity: 90.35%, AUC: 0.829±0.025).

**Table 4:** Coefficients of the Logistic Regression Model (N = 927).

	Coefficient (β)	SE	OR	95% CI	p value
NG tube	−2.594	0.317	13.389	7.193–24.924	<0.0001
Steroids	−1.975	0.415	7.207	3.195–16.256	<0.0001
Foley catheter	−1.812	0.282	6.125	3.524–10.645	<0.0001
Hemodialysis	−1.527	0.528	4.606	1.637–12.958	0.004
Stress ulcer prophylaxes	−1.368	0.303	3.928	2.171–7.108	<0.0001
Arterial line	−0.950	0.432	2.586	1.109–6.032	0.028
CVC	−0.753	0.374	2.123	1.021–4.415	0.044
Constant	6.518	0.754	-	-	-

Abbreviations: SE = standard error, OR = odds ratio, CI: confidence interval, CVC = central venous catheter, NG = nasogastric.

### Detection of Infection by ANN

**Figure 1:**
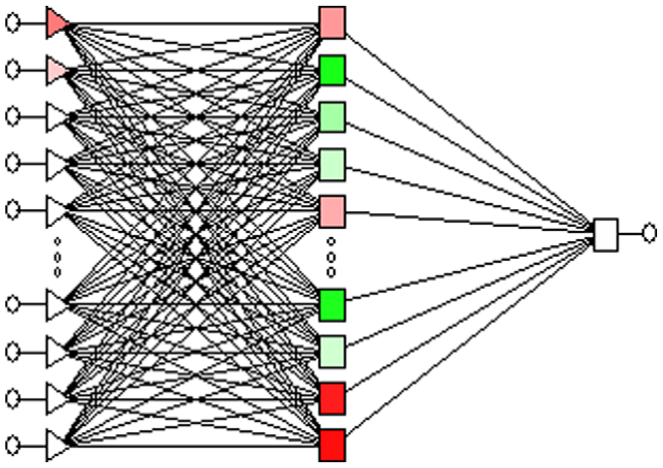
The Optimal Network Architecture of The Artificial Neural Network. A multilayer perceptron with 16 input nodes and 13 hidden nodes in the network.

**Figure 2:**
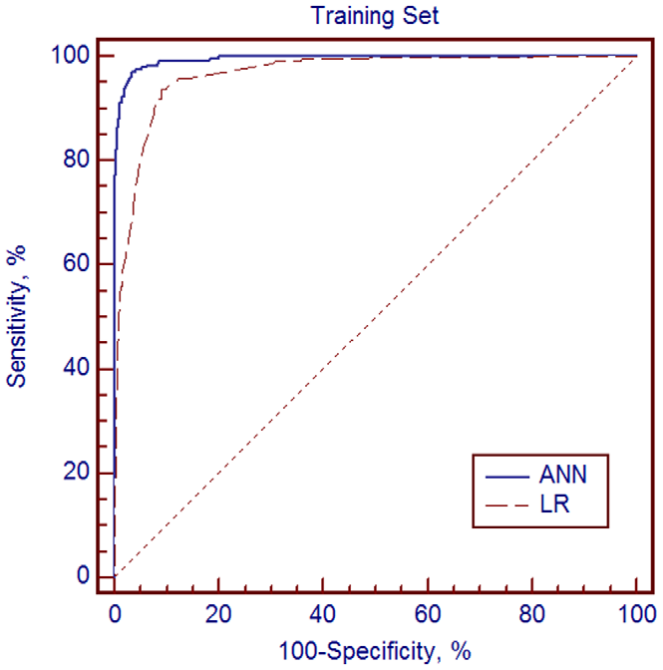
Comparison of The Area Under the Receiver Operating Characteristic Curves (AUCs) in Training Set (N = 927). All variables were included in artificial neural network (ANN) model and 7 variables were included in logistic regression (LR) model. The AUCs for ANN and LR are 0.995±0.003 and 0.966±0.008 respectively (p<0.001).

**Figure 3:**
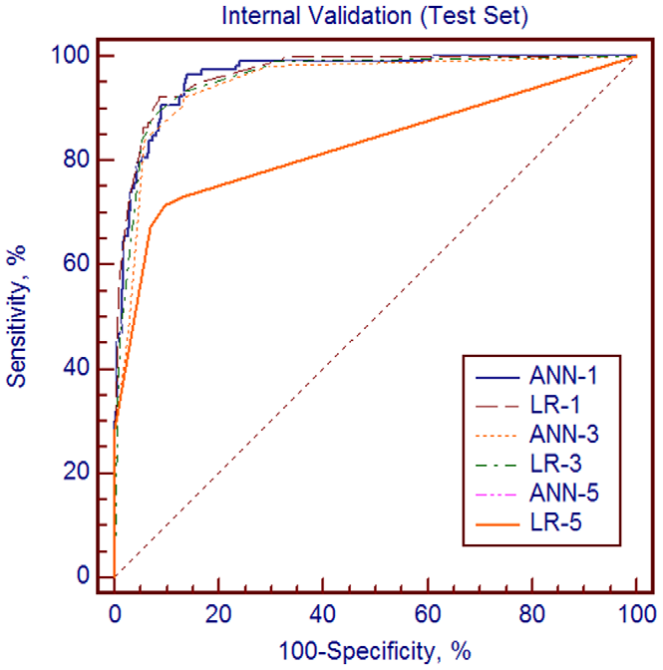
Comparison of The Area Under the Receiver Operating Characteristic Curves (AUCs) in Internal Validation Set (N = 461). The comparison of AUCs between different variable groups of artificial neural network (ANN-1, ANN-3, ANN-5 for Group 1, 3, 5 respectively) and logistic regression (LR-1, LR-2, LR-3 respectively) in internal validation.

The first model showed that the multilayer perceptron network with 16 input nodes and 13 hidden nodes provided the optimal network architecture (figure 1) which also gave excellent performance (accuracy: 95.04%; sensitivity: 97.06%; specificity: 96.52%), the AUC outperformed which of LR (0.995±0.003 versus 0.966±0.008, p<0.001) in training set (figure 2). Then other ANN models using different group were analyzed as it was done with LR. The results in test set also displayed good performance irrespective of inclusion or non-inclusion of medical devices as variables (accurate rate: 90.51% in training set and 91.54% in test set in Group 3; accurate rate: 85.33% in training set and 85.47% in test set in Group 5). The results of LR in comparison with ANN in training set and internal validation were shown in table 5, 6. Comparing these two algorithms, as studies showed, ANN performed better than LR just in the beginning. Later, the differences became less significant as variables decreased in later “variable rotation” steps. In terms of internal validation, there were no statistical significances between ANN and LR in the performance of detection in different groups of variables (p = 0.507, 0.574, 0.095, 0.553 for Group 1 to 4 respectively) (table 6 and figure 3). Interestingly, in Group 5, we can get the same AUC for ANN and LR in both sets (0.867±0.016 in training set; 0.829±0.025 in test set, p = 1.000) as shown in table 5 and 6.

**Table 5:** Comparison of ANN and LR in Training Set, % (N = 927).

Models	Accuracy	Sens.	Spec.	PPV	NPV	AUC^a^	p value^b^	p value^c^	p value^d^
Group 1									
ANN	95.04	97.06	96.52	90.6	99.0	0.995±0.003	-	-	
LR	91.05	93.7	91.0	78.2	97.7	0.966±0.008	<0.001		-
Group 2									
ANN	89.97	92.44	89.7	75.6	97.2	0.949±0.010	-	<0.001	
LR	89.32	92.44	89.7	75.6	97.2	0.947±0.010	0.796		0.005
Group 3									
ANN	90.51	91.60	87.37	71.5	96.8	0.948±0.010	-	<0.001	
LR	90.40	90.34	89.40	74.7	96.4	0.953±0.010	0.183		0.054
Group 4									
ANN	86.62	85.29	85.78	67.4	94.4	0.884±0.015	-	<0.001	
LR	86.73	84.45	86.65	68.6	94.2	0.886±0.015	0.490		<0.001
Group 5									
ANN	85.33	82.35	86.36	67.6	93.4	0.867±0.016	-	<0.001	
LR	85.33	82.35	86.36	67.6	93.4	0.867±0.016	1.000		<0.001

a Mean±SE,

b comparison with same variables set,

c comparison with ANN model,

d comparison with LR model.

Group 1: all variables.

Group 2: high odds ratio variables (Foley, nasogastric tube and steroids).

Group 3: medical devices as variables (Foley, CVC catheter, arterial line and nasogastric tube).

Group 4: low odds ratio variables (CVC catheter, arterial line and stress ulcer prophylaxes).

Group 5: underlying condition and medications as variables (hemodialysis, stress ulcer prophylaxes and steroids).

Abbreviations: ANN = artificial neural network, LR = logistic regression, Sens. = sensitivity, Spec. = specificity, PPV = positive predictive value, NPV = negative predictive value, AUC = area under the receiver operating characteristic curve.

**Table 6:** Comparison of ANN and LR in Internal Validation, % (N = 461).

Models	Accuracy	Sens.	Spec.	PPV	NPV	AUC^a^	p value^b^	p value^c^	p value^d^
Group 1									
ANN	90.24	96.64	85.96	70.6	98.7	0.964±0.012	-	-	
LR	91.54	92.44	91.52	79.1	97.2	0.969±0.011	0.507		-
Group 2									
ANN	90.02	90.76	86.55	70.1	96.4	0.949±0.014	-	0.177	
LR	87.64	90.76	86.55	70.1	96.4	0.952±0.014	0.574		0.024
Group 3									
ANN	91.54	92.44	86.84	71.0	97.1	0.949±0.014	-	0.205	
LR	91.76	96.64	85.96	70.6	98.7	0.959±0.013	0.095		0.295
Group 4									
ANN	86.98	81.51	89.18	72.4	93.3	0.873±0.022	-	<0.001	
LR	87.64	80.67	90.06	73.8	93.1	0.876±0.022	0.553		<0.001
Group 5									
ANN	85.47	71.43	90.35	72.0	90.1	0.829±0.025	-	<0.001	
LR	85.47	71.43	90.35	72.0	90.1	0.829±0.025	1.000		<0.001

a Mean±SE,

b comparison with same variables set,

c comparison with ANN model,

d comparison with LR model.

Group 1: all variables.

Group 2: high odds ratio variables (Foley, nasogastric tube and steroids).

Group 3: medical devices as variables (Foley, CVC catheter, arterial line and nasogastric tube).

Group 4: low odds ratio variables (CVC catheter, arterial line and stress ulcer prophylaxes).

Group 5: underlying condition and medications as variables (hemodialysis, stress ulcer prophylaxes and steroids).

Abbreviations: ANN = artificial neural network, LR = logistic regression, Sens. = sensitivity, Spec. = specificity, PPV = positive predictive value, NPV = negative predictive value, AUC = area under the receiver operating characteristic curve.

### Prediction of Infection by Scoring System

The equation for the prediction of HAI derived from LR is:

*Logit (odds of HAI) = −4.4622+2.5499[NG tube]+1.8124[Foley]+0.9502[A-line]+0.7528[CVC]+1.9751[Steroids]+1.3682[Stress-ulcer prophylaxes]+1.5272[Hemodialysis]*

*Where [variable] = 1 if the patient presents with the character and 0 otherwise.*

The probability of HAI = e^logit^/(1+e^logit^)

In order to obtain the simplest weights, we set the coefficient of CVC as the denominator and rounded the proportions as the weights of the variables.

After logistic transformation, the shrink equation of the scoring is:

*Total HAI Score = 3[NG tube]+2[Foley]+1[A-line]+1[CVC]+3[Steroid]+2[Stress-ulcer prophylaxes]+2[Hemodialysis]*

*Where [variable] = 1 if the patient presents with the character and 0 otherwise.*

The optimal cut points for predicted values were 3, 2 and 1 for Group 1, Group 3 and Group 5 respectively. That is, Score >3 in Group 1 indicates infection. The performances of scoring of Group 1 both for training set (accuracy: 91.26%; sensitivity: 94.12%; specificity: 90.28%) and internal validation (accuracy: 90.67%; sensitivity: 91.60%; specificity: 90.35%) were excellent. Using medical devices as variables only (i.e. Group 3) also displayed comparatively good performances with high accuracy (89.53% in training set and 88.50% in internal validation), the mean values of AUC were 0.953±0.010 and 0.958±0.013 for training and internal validation sets respectively as seen in LR. Using only three variables of underlying condition and medications (i.e. Group 5) also resulted in good prediction rates in internal validation (accuracy: 83.30%; sensitivity: 73.11%; specificity: 86.84%, AUC: 0.815±0.025). In comparison with LR, there is no statistical significant in terms of discrimination in training and internal validation.

### Comparison of Predictive Performance on External Validation

**Figure 4:**
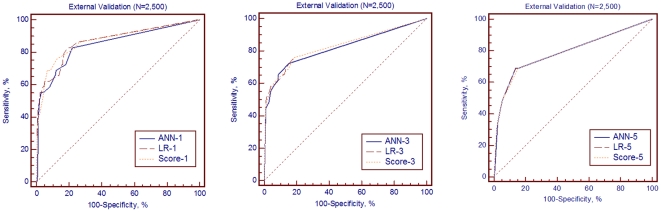
Comparison of The Area Under the Receiver Operating Characteristic Curves (AUCs) in External Validation Set (N = 2,500). The comparison of AUCs between different variable groups of artificial neural network (ANN-1, ANN-3, ANN-5 for Group 1, 3, 5 respectively), logistic regression (LR-1, LR-2, LR-3 respectively) and scoring system (Score-1, Score-3, Score-5 respectively) in external validation.

Out of 2,500 admitted patients at Far Eastern Memorial Hospital, a 1,200-bed academic tertiary teaching center, 1,161 (46.6%) were female patients with the mean age of 52.32±16.11 years (range 17 years–80 years). Twenty-night patients (1.2%) who met the diagnostic criteria of CDC were collected by infection control professionals as infection group. Good performance could be obtained from Scoring, LR and ANN (AUC: 0.871±0.043, 0.870±0.043 and 0.850±0.045, respectively). The overall accuracy, sensitivity, specificity and AUC of each model for variable groups are shown in table 7 and figure 4. The results indicated that using different variable combination as predictive models could be applied on an external independent population.

**Table 7:** Comparison of ANN, LR and Scoring in External Validation, % (N = 2,500).

Models	Accuracy	Sens.	Spec.	AUC^a^	p value^b^
Group 1					
ANN	96.12	82.76	78.15	0.850±0.045	-
LR	98.76	82.76	80.90	0.870±0.043	0.447
Score	91.24	68.97	91.50	0.871±0.043	0.362
Group 3					
ANN	95.44	72.41	84.66	0.820±0.048	-
LR	98.52	75.86	81.63	0.831±0.047	0.521
Score	92.24	62.07	92.59	0.830±0.047	0.524
Group 5					
ANN	94.28	68.97	86.16	0.791±0.050	-
LR	98.84	68.97	86.16	0.792±0.050	0.929
Score	84.44	68.97	84.62	0.791±0.050	0.967

a Mean±SE,

b comparison with ANN model.

Group 1: all variables.

Group 3: medical devices as variables (Foley, CVC catheter, arterial line and nasogastric tube).

Group 5: underlying condition and medications as variables (hemodialysis, stress ulcer prophylaxes and steroids).

Abbreviations: ANN = artificial neural network, LR = logistic regression, Sens. = sensitivity, Spec. = specificity, AUC = area under the receiver operating characteristic curve.

## Discussion

The scoring system, with ANN and LR developed excellent prediction models for HAI form EHR. The ANN showed no statistical significance for all variable combinations compared to LR. The discriminatory power of both models was comparable with previous study [15].

On August 1, 2007, The Centers for Medicare and Medicaid Services (CMS) announced that it will not pay for few HAIs, including catheter-related urinary tract infection and vascular catheter-related infection [25], because some of these infections are common, expensive, and “preventable”. Such rules have not been applied in Taiwan or some other countries yet, but it will be soon regarded as an important principal for the reimbursement and benchmarking.

There are several types of device-associated infection (DAI) such as CVC-associated infection, or catheter-related bloodstream infection (CRBSI), catheter-related urinary tract infection (CAUTI), and ventilator-associated pneumonia, VAP [7]. The prevalence varies by settings and countries. A Turkish survey in 13 medical-surgical ICUS from 12 hospitals, all members of International Nosocomial Infection control Consortium (INICC), the definitions of the US Centers for Disease Control and Prevention National Nosocomial Infections Surveillance System (NNISS) were applied, reported an overall rate of 38.3% or 33.9 DAIs per 1,000 ICU-days. VAP (47.4% of all DAI, 26.5 cases per 1,000 ventilator-days) gave the highest risk, followed by CRBSI (30.4% of all DAI, 17.6 cases per 1,000 catheter-days) and CAUTI (22.1% of all DAI, 8.3 cases per 1000 catheter-days) [26], while NNIS report of US ICUs (1992–2004) reported overall rate of CVC was 4.0 per 1,000 CVC-day, 5.4 per 1,000 ventilator-day for VAP and 3.9 per 1,000 catheter-day for CAUTI in ICUs of teaching hospitals [27]. ICU is not the only place where DAI is reported, many CVCs are also used outside the ICU, and the rates of CRBSI in these settings appear to be similar to that of the infections in ICUs [28]. A German study revealed that in non-ICU patients, the device-associated HAI rates were 4.3 per 1,000 CVC-days for CVC-associated bloodstream infections and 6.8 infections per 1,000 urinary catheter–days for catheter-associated urinary tract infections [29].

The DAIs attribute to HAI and cause high morbidity and mortality, nerveless prolonged stay and high expense is consequential. Another German study conveyed by Kamp-Hopmans et al. found that the risk factors contributing HAI in surgical wards were: RR of enteral tube feeding over 48 hours was 6.6 (95% CI: 3.2–7.9) followed by ventilation used over 24 hours of 5.0 (95% CI: 3.2–7.9) and used of steroids of 3.4 (95% CI: 2.0–6.0) for respiratory infection; urinary catheter used for UTI was 3.9 (95% CI: 1.7–9.0) [30].

The current reimbursement system fails to penalize hospitals for largely preventable conditions due to medical negligence. The system rewards them in the form of special reimbursement. As the CMS wishes, hospitals should additionally enhance their efficiency in preventing the preventable adverse events and reduce the supposed expenses to be reimbursed priory in the future. On the other hand as our results indicated, to monitor and predict the possibility of HAIs before infection would contribute to reduce the unintended consequences and expenses for such complications [31]. As more information becomes available electronically in the healthcare setups, the use of highly reliable electronic surveillance for HAIs has become effective in daily usage, some significant progress is being made for surveillance of CRBSI, VAP, and other HAIs [32].

Our results show the high accuracy of prediction with scoring and both models. From the analyses of LR, we found 7 risk factors relevant to HAI, in which Foley and CVC were included. As we anticipated, the results are quite compatible to that of previous studies and, explore new insights of factors. Medical devices are examples for us to review the role in predicting HAI. The study revealed the differences, with or without presence of these devices as main parameters. No matter how much information is available, we can accurately predict HAI with simple parameters. We have also found the factors that proved to be significant than the HAI by medical devices alone.

Ample evidence shows that invasive devices contribute to the occurrence of HAI, interestingly, NG tube being less invasive but contribute more that the odds ratio ranks first in this study. NG tube feeding is known to be a significant cause of aspiration pneumonia in critical patients due to the gastroesophageal reflux of bacteriologically contaminated gastric contents and subsequent microaspiration of these contents to the lower airways. The NG tube in ventilated patients is partially responsible for reflux and has been recognized as a risk factor for nosocomial pneumonia [33], [34].

Patients on hemodialysis are at particular risk for HAIs because of frequent hospital admissions and numerous comorbid conditions such as uremic toxicity, and anemia of chronic renal failure. All pre-existing conditions contribute to an immunocompromised state [35], and patients on hemodialysis are frequently exposed to invasive devices, especially vascular access [36]. Study shows that a greater index of comorbidity was significantly associated with HAIs among the chronic hemodialysis population. Urinary tract infection was the most common infection in this study because although UTI may present with decreased urine output [35], the clinical suspicion of oliguria as UTI is understandably low in patients on dialysis. Bloodstream infection is another major cause of morbidity in patients receiving hemodialysis. Hemodialysis access through arteriovenous fistula was associated with the lowest risk for BSI. The relative risk for infection was 2.5 with arteriovenous graft access, 15.5 with cuffed and tunneled CVC access, and 22.5 with uncuffed CVC access in a Canadian study [36].

A large scale epidemiologic survey showed that all the protocol of stress ulcer prophylaxis exhibits increased risk of pneumonia in ICU patients [5]. It is considered to be the effect of increase in gastric pH in association with an increased risk of VAP.

However, evidence suggests that only VAP (and not any other HAIs) was related to the use of stress ulcer prophylaxis. Our result is compatible to major studies indicating the impact of stress ulcer prophylaxes on the incidence of HAIs (adjust OR: 4.403; 95% CI: 1.981–9.787).

The use of glucocorticosteroids is correlated to HAI, mostly with pneumonia being the most common. The host is susceptible to increased risk of infection due to immunosuppressive effect of steroids involving release of cytokines and other anti-inflammatory mediators. In our study, we found that systemic steroidal therapy plays an important role in contributing HAIs, and was compatible with other studies [30], [37].

Using medical devices as variable combination for predicting HAI is a significant finding of this study. Efforts can be made to prevent consequent infection. If indwelling device is needed, for examples, one should choose antimicrobial coated NG tube or vascular devices to avoid aspiration pneumonia and bloodstream infection, respectively [38]. To mitigate HAI, early device removal or using alternative procedure is the probable solution.

We applied different combinations of variables in detecting HAIs using both ANN and LR models and even developing a simple scoring system, and results were significant. Such variable sets could be used in different clinical settings according to the ease of information retrieval. For most clinical scenarios, medical devices usage is recognized easily by observation only, it is convenient to detect and predict the occurrence of HAI without collecting other clinical information which the hospital information system (HIS) has not been well established. From the administrative point of view, on the other hand, underlying clinical condition and therapeutic agents given to patients could be accessed by way of EHR or HIS instead of traditional chart review, which allows clinicians in decision making in preventing HAI without seeing patient personally.

The development of the scoring system is the most significant result of this study that variables are mutually exclusive but can be put together as predictive parameters. Like other medical scoring system, the usefulness of this scoring is the simple calculation using limited parameters. Although the numeric range of the scores ranks between 0 and 14, sum of equation over 3 predicts infection, a calculation easily performed by one's fingers. In infection surveillance, microbiology report are considered the most important initial source of information in screening for infection followed by patient's chart, admitting office, house staffs, discharge summary, kardex, fever chart, antibiotic orders and quality assurance personnel [39]. An important issue lies in distinguishing between colonization and infection, the latter representing invasion whereas the former indicates only an uneasy truce. This is important as urinary catheter-induced positive urine culture largely determined the presence or absence of “infection”. Patients with noninvasive colonization do not require antimicrobial treatment, but may require careful regulation of fluid balance and diet to ensure adequate urine output and pH value. If the diagnosis of infection was based purely on microbiology reports without reference to the patients' condition, then the incidence will be overestimated and misinterpreted. The number of infection identified depends on the intensity of surveillance; however, the intensity of surveillance depends on having adequately well trained infection control personnel. The surveillance works effectively with well-developed system. If this scoring system can be used for screening candidates of HAI at the stage of information collection before going to bedside for suspect cases enrollment, the infection control personnel and physicians can contribute more efforts in preventing HAI instead of monitoring only. The system may benefit for more large-scale hospitals and should not be a complex calculation that makes clinicians more reluctant to use in their busy daily works. But we should always keep in mind that the importance of such individual prognostication lies in the clinical judgment instead of the issue of calculation [21].

In 2009, The US government (Department of Health and Human Services [HHS] Office for Civil Rights, HHS Centers for Medicare and Medicaid Services and HHS Office of the National Coordinator for Health Information Technology) released statutorily required regulations under the Health Information Technology for Economic and Clinical Health (HITECH) Act provisions that included in the American Recovery and Reinvestment Act (ARRA) which addressed breach notification requirements for protected health information, Medicare and Medicaid incentives for meaningful use of EHR. These regulations build on the framework and financial support authorized under ARRA for increased use of EHR and enhanced privacy and security provisions for protected health information. The passage of ARRA significantly changed the regulatory landscape by authorizing substantial financial and technical support for the adoption and the use of EHRs and enhancing information privacy and security requirements [40]. As the ARRA project has been released, the EHR will be implemented in nearly every healthcare facility including small and rural hospitals. Therefore, the ability of information management will become easier by data mining or other computational tools. Using simple scoring system, physicians can just rely on mental arithmetic in predicting HAI today, however, HITECH encourages the adoption and use of HER and automatic computation can be applied for even real-time surveillance in order to improve patient safety in the future.

There are certain limitations of this study. The scoring system derived in this study is based on an available hospital data set, due to the ever-changing landscape of HAI, researchers may consider using more current or local data set to fine-tune the scoring system before putting into large-scale use. Secondly, the concept of ANN seems to be attractive but neural networks are not analyzed easily based on risks attributable to specific clinical characteristics or statistical significance because a neural network relies on its internal representation of weights and functions to process data instead of simple and clear equations like a regression model [41], intentionally there is no comparison between discriminatory power of ANN and LR. We observed the advantages of both models in different stages of this study. Thirdly, we only registered the patients between the ages of 16 to 80; hence, we could not realize and categorize the conditions between pediatric and geriatric populations. Fourthly, we pooled the patients from ICUs and non-ICU wards, and all HAIs were regarded as one kind of infection, which may overestimate the prediction probability towards high incident infection type, such as UTI. Further analysis should be made in order to understand the detailed information about the different type of infections and impacts on critically ill patients. Furthermore, the laboratory testing reports and patients' vital data were note included due to unavailability of EHR at the time of data collection. Some of this information are relevant to HAIs and should be considered in the future. The EHR system may not be implemented in every hospital, but as the release of ARRA-HITECH, it will become popular afterwards. Taking the advantage of EHR, variables could be used as many as possible to make more precise prediction since the data retrieval is not a difficult task. Lastly, human and environmental factors that lead to HAIs were not evaluated. Washing hands, laundering of white coats, not wearing a tie [42], might contribute to improve HAIs and promise further investigations.

In conclusion, our study developed accurate scoring system in predicting HAI with simple parameters with discrimination, and validated the system by ANN and LR that could be the foundation for computation in the future. Using parameters either by observation of medical devices used or data obtained from EHR also provided satisfactory excellent prediction outcome, which can be utilized in different clinical settings by ease of information retrieval. It also can be used as a simple measure to reduce HAI incidence in the hospital.
